# Prevalence and correlates of probable anxiety and depression among young people: A subnational cross-sectional study in Ghana

**DOI:** 10.1017/gmh.2026.10270

**Published:** 2026-07-07

**Authors:** Joyce Aputere Ndago, Emmanuel Asampong, Franklin N. Glozah, Faustina Hayford Blankson, Philip Teg-Nefaah Tabong, Adanna Uloaku Nwameme, Philip Baba Adongo, Benedict Weobong

**Affiliations:** 1 Department of Social and Behavioural Sciences, University of Ghana, Ghana; 2 Department of Social and Behavioural Change, University for Development Studies, Ghana; 3 School of Global Health, York University Faculty of Health, Canada

**Keywords:** depression, anxiety, suicide, children, young people, ecological zones and Ghana

## Abstract

We assessed the prevalence of probable depression and anxiety among young people in Ghana to inform evidence-based responses to youth mental health needs. This subnational high-risk population survey was conducted among 602 young people (YP) aged 13–24 years across three ecological zones of Ghana. Screening was done using the Patient Health Questionnaire-9 (PHQ-9) and Generalized Anxiety Disorder (GAD-7) tools. Demographic, social and economic indicators were assessed. Logistic regression was used to generate adjusted relative risk (aRR) with 95% confidence intervals (CIs). There was evidence of probable depression and anxiety among participants surveyed. In total, 8.6% (95% CI, 6.5–11.2) of the participants met criteria for probable depression, 5.9% (95% CI, 4.2–8.2) for probable anxiety and 2.8% (95% CI, 1.6–4.5) for comorbid depression and anxiety (cDA). Correlates of cDA included living in a household enrolled in two or more social protection programs (aRR: 3.70; 95% CI, [1.00–13.66]; anxiety: 3.63 [1.57, 8.4]; depression:1.93 [1.00, 3.71]) and being currently in school (aRR: 2.82; 95% CI, 0.78–10.10). The burden of probable depression or anxiety evidenced in this study underscores the need to integrate mental health promotion and screening into existing social protection schemes and school-based interventions to enhance early detection and support for youth mental health in Ghana and similar LMIC settings.

## Impact statement

This manuscript offers evidence from a subnational high-risk population study on the prevalence and correlates of probable anxiety and depression among young people in Ghana. It highlights mental health as a growing yet under-recognized public health concern in Sub-Saharan Africa. By revealing how socioeconomic disadvantages, school environment and social support affect youth mental well-being, the research advances understanding of specific factors driving psychological distress in low-resource settings. The findings underscore the importance of context-specific, focused mental health strategies that incorporate routine screening, psychological support and community-based interventions within Ghana’s health and education systems. These lessons can serve as a guide for other lower-middle-income countries seeking to reform mental health services for young people.

## Introduction

Adolescent mental health issues are emerging as a significant public health concern across many low- and middle-income countries, particularly in Africa (Tinsae et al., 2024). Depression and anxiety are the two most burdensome mental health conditions that young people experience (WHO, 2021, 2022). The COVID-19 pandemic increased the global prevalence of depression and anxiety by 25% (Racine et al., 2021), with children and adolescents disproportionately affected (Ma et al., 2021; Racine et al., 2021; Robinson et al., 2022). According to the 2019 national Global Burden of Disease study, the top 10 contributors to the burden of disease among young people in Ghana and SSA include mental health conditions, which rank in the top 2 (Global Burden of Disease, 2022; Weobong et al., 2024). This may be attributed to heightened socioeconomic and social disadvantage (Glozah et al., 2018) and the poor state of mental health services in primary healthcare. It is important to conduct prevalence studies for two main reasons: first, to assess the need; and second, to provide baseline data.

However, the evidence on the burden of mental health conditions in this demographic is patchy due to the paucity of epidemiological research in LMICs (Awenva and Read, 2010; Mwangi et al., 2023). We identified only two systematic reviews on the topic; one provided a synthesis of the prevalence of mental distress (undifferentiated manifestations of anxiety and depression symptoms) in Africa (Tinsae et al., 2024) (*n* = 18 studies, none from Ghana); the other focused on studies in South Asia (Mudunna et al., 2025) (*n* = 117 studies). Very few of these studies had been conducted in community or population settings; almost three-quarters of the studies from South Asia (India and Pakistan) were school-based, and only seven of the studies in Africa (Ethiopia, Tanzania, Uganda, South Africa, Liberia, Nigeria, Benin, Zambia, Mozambique and Morocco) were population-based (Jorns-Presentati et al., 2021; Ghosh, 2023; Atewologun et al., 2025). The prevalence of mental distress among adolescents in Africa was 27% (Jorns-Presentati et al., 2021; Sequeira et al., 2022). In South Asia, the prevalence of depression ranged from 21% to 98% in school-based and 18% to 88% in nonschool-based settings. Determinants were largely similar across these two regions: bullying, victimization and the experience of hunger. In the Asian region, poor peer relationships and substance use/abuse were important determinants. Higher education was protective of mental well-being. None of the reviews included country-wide studies.

There is a general lack of epidemiological studies on this topic in Ghana, and the few published ones have been limited to specific regions and subgroups and have been conducted in school-based settings. One of these studies, conducted in the south of Ghana among in-school young people, estimated a depression prevalence of 38% (Obeng-Okon et al., 2024). In the northern sector, the prevalence of anxiety was up to 74% (Amadu et al., 2024). Such epidemiological studies are a research priority in Ghana (Weobong et al., 2022). There is also evidence that depressive and anxiety symptoms frequently overlap, but these comorbidities are less studied among young people (Melton et al., 2016).

Studies that employ country-wide approaches, such as national surveys, are useful for generating generalizable country prevalence estimates that are critical for planning mental health services. National surveys are limited, particularly in LMICs, due to the resource-intensive requirements that characterize the conduct of these studies. Proxy national surveys, such as subnational cross-sectional surveys, have the potential to bridge this gap. The Global School-based Health Surveys (GSHSs) (WHO, 2021, 2022), which includes Ghana, adopts a national survey design, but the data are only generalizable to in-school adolescents and do not include standardized measures for depression and anxiety.

In this article, we present estimates of the burden of probable depression, anxiety and factors associated with this burden in a high-risk population-based subnational cross-sectional study of young people across three ecological zones of Ghana. This study was part of a global children and youth-led participatory research initiative coordinated by UNICEF – ‘the Children & Young People Participatory Research and Communication for Change Initiative’. The multicountry initiative aimed to explore how to increase young people’s (13–24 years) involvement in shaping solutions for global challenges in the context of COVID-19.

## Methods

### Design

This was a subnational cross-sectional study of young people across three ecological zones of Ghana nested within ‘the Children & Young People Participatory Research and Communication for Change Initiative’. This was a UNICEF-coordinated, global, children- and youth-led participatory research initiative. The multicountry initiative aimed to explore how to increase young people’s (13–24 years) involvement in shaping solutions for global challenges in the context of COVID-19. Young people were engaged as partners in research and decision-making on critical topics such as infectious diseases, mental health and climate emergencies. In Ghana, the initiative was undertaken in partnership with the Department of Social and Behavioural Sciences, School of Public Health, University of Ghana.

Ethical approval for the study was granted by the ethics review committee of the Ghana Health Services (GHS-ERC:03/01/22). The study was conducted in March 2022.

### Setting and participants

Participants were selected using multistage random sampling techniques, based on geographic location within each of the three ecological zones of Ghana: Southern, Middle and Savannah. In total, 16 districts (Bole, Gonja, Wa East and West, Banda, Wenchi, Kwahu Afram Plains North, Kwahu South, Kumasi Metro, Obuasi, Biakoye, Jasikan, Bodi, Bia, Ayawaso West Wuogon and Korle Klottey) were selected from eight regions (Savannah, Upper West, Bono, Eastern, Ashanti, Oti, Western North and Greater Accra) within the three zones. Specific study sites and participants within the 16 districts were selected based on any of the following criteria: aged 13–24 years; willingness to commit 1 hour; and belonging to a marginalized group such as low-income groups, ethnic and racial minorities, displaced people, rural populations, urban ghettoes, girls and children with disability.

#### Selection of CYP researchers

With the support of a local research organization (JMK Consulting Ltd) with extensive reach across Ghana, 30 young people aged 18–24 years pursuing undergraduate program at the University of Ghana with research interest were invited to form the research team (enumerators). Enumerators were purposively selected from across the 16 regions of Ghana, including the requirement that they reside in these regions. Enumerators were trained over 3 days to gain competencies in the following areas: basics of mental health; introduction to research; ethics of research; the research process; and understanding and sharing findings. During training, the enumerators were assessed on pre- and post-knowledge using a questionnaire, performance in the training and demonstration of the interview at the end of the training. Our quality assurance approach followed the example of our previous study (Ae-Ngibise et al., 2023). Training on the English version of the measures was conducted by BW and PAB. Experienced bilingual (English and the predominant local language in the study districts) data collectors were trained in a 3-day workshop on the content and administration of all study questionnaires, including the qualitative interview. This involved forward translation to the local languages and back translation, particularly paying attention to key constructs in the back translation. Consensus on the translation of each item was obtained. Only enumerators who attained competence were recruited to conduct the interviews.

#### Selection of CYP respondents

Eligible young people were selected using a multistage random sampling technique. We estimated a total sample size of 600 young people would provide a precision of ±4.3% around the estimated prevalence based on the following assumptions: based on a previous estimate of 38% as the prevalence for depression among school-going young people in Ghana (Obeng-Okon et al., 2024), we opted for a conservative 20% observed prevalence of comorbid depression and anxiety; design effect (DEFF) of 1.8 (accounting for clustering around the three ecological zones). Potential respondents were selected based on their location within each of the three ecological zones, following these steps. First, in each of the three ecological zones, two deprived regions (*Deprived areas are locations lacking essential resources, services or opportunities, such as inadequate housing and lack of economic opportunities, which result in poverty and social disadvantages*) were randomly selected. Following this, two districts in each of the two regions were randomly selected (*n* = 4 districts). Next, 10 enumeration areas (EAs) in each of the 4 randomly selected districts were also randomly selected (*n* = 40 EAs). Subsequently, 5 communities in each of the 10 EAs were randomly selected (*n* = 20 communities). Five young people in each of the 20 communities were selected using systematic random sampling techniques and interviewed. On reaching the selected community, the young researcher identified a location and spun a bottle to select the first house. Subsequent houses were selected from this point using a fixed sampling interval of every second house in a straight line until reaching the boundary of the community. One respondent each was selected from the randomly selected households in a given community. Where there was more than one eligible young person in each household, the Kish selection grid (1949) (Németh and Hungary, 2001)was used to select only one respondent. Given the nested design, this sampling strategy was not designed to achieve national representativeness, but rather to ensure that the perspectives of higher-risk and often underrepresented groups were meaningfully captured.

#### Data collection

The study employed a youth-led qualitative and quantitative research methodology for data collection. This article presents results only for the quantitative strand of the study. Data were collected by trained youth researchers (*N* = 30) using tablet computers and audio recorders. The assessments of depression and anxiety were made by administering the English version of the PHQ-ADS (Kroenke et al., 2016) with flexibility to administer the local-language translated versions of the measures at the point of the survey. This combines the 9-item Patient Health Questionnaire (PHQ-9) and the 7-item Generalized Anxiety Disorder scale (GAD-7). It is a 16-item measure and can be scored on a continuous scale with scores ranging from 0 to 48, with higher scores indicating higher levels of depression and anxiety symptomatology. The PHQ-ADS is considered a reliable and valid composite tool for evaluating two of the most prevalent mental conditions in both clinical practice and research (Kroenke et al., 2016). The construct validity of the PHQ-9 and GAD-7 among adolescents in Ghana has been previously reported (Anum et al., 2019). Criterion validity among adolescents in Ghana has not been examined and reported; however, a study in South Africa reports a cutoff of 10+ as indicative of moderate to severe depression (Marlow et al., 2023). This cutoff was used in the present study for both the PHQ-9 and GAD-7.

### Outcome measure

The primary outcome is the estimated prevalence of probable comorbid depression and anxiety, operationalized as simultaneous scores above 9 on both the PHQ-9 and GAD-7 screening tools. This is the most used cut point on both the PHQ-9 and GAD-7 screens for comorbid depressive and anxiety conditions (Kroenke et al., 2016). Both measures evaluate the presence and severity of symptoms on a 4-point Likert scale over the past 2 weeks, adhering to Diagnostic and Statistical Manual criteria (Kroenke et al., 2001). The scoring ranges from 0 to 27 for the PHQ-9 and 0 to 21 for the GAD-7, with higher scores indicating greater symptom severity. Secondary outcomes were reported separately for the PHQ-9 and GAD-7.

### Potential determinants

#### Sociodemographic information

Age of young person at recruitment, sex, education, employment status, school-going status and whether the young person’s household is currently benefiting from a social protection program. Others included the ecological zone of residence.

#### Analysis

Primary outcome prevalence was estimated by creating a binary variable: simultaneous scores above 9 on both the PHQ-9 and GAD-7 coded as 1 (caseness criterion). Secondary outcome prevalence was estimated by creating a binary variable: scores above 9 on the PHQ-9 (caseness for probable depression) and GAD-7 (caseness for probable anxiety). Logistic regression was used to examine the association between potential determinants and the primary and secondary outcomes. We followed a similar approach used in our previous studies (Weobong et al., 2014). First, we assessed the association of each sociodemographic/socioeconomic factor. All factors associated with *p* < 0.1 in the univariate models were included, together with sex and age, in a multivariable regression model at the first stage. Factors that remained statistically significant predictors of comorbid depression and anxiety at the *p* < 0.05 level were noted. At the second and final stage, a multivariable model was fitted with only those that remained significant, together with sex and age. This was repeated for the secondary outcomes. Effect sizes are reported as crude and adjusted relative risks (aRRs) estimated using the marginal standardization technique with 95% confidence intervals estimated *via* the delta method (Localio et al., 2007). Analyses were conducted using STATA 18.

## Results

### Description of study sample

Table 2 shows the sociodemographic/socioeconomic characteristics of the study population. The results show an almost equal gender distribution, with 49.8% and 50.2% of males and females in the study, respectively. The modal age group was 20–24 years (43.9%). Almost half of the sample population had Basic education (47.2%). A little over two-thirds (67.1%) were currently in school, and 51.3% were in households that had enrolled in at least one social protection program. There was an almost equal distribution of young people from each of the ecological zones.

Among the 602 young people, 2.8% (*n* = 17) (95% CI, 1.6–4.5%) experienced probable comorbid depression/anxiety; 8.6% (*n* = 52) (95% CI, 6.5–11.2%) reported probable depression and 5.9% (*n* = 36) (95% CI, 4.2–8.2%) met the criterion for probable anxiety. Table 1 provides a summary of the severity of study outcomes based on the severity norms of: none, mild, moderate, moderately severe and severe. We note a high prevalence of comorbid mild and moderate depression (17%) in this sample of young people.

**Table 1.** Severity of mental health outcomes Table 1. long description.

Severity of mental health outcomes			
Severity category	Depression (*N* = 602)*n* (%)	Anxiety (*N* = 602)*n* (%)	Comorbid depression-anxiety (*N* = 602)*n* (%)
None	312 (51.3)	392 (65.1)	259 (43.0)
Mild	238 (39.5)	174 (28.9)	98 (16.3)
Moderate	40 (6.6)	35 (5.8)	9 (1.5)
Moderately severe	11 (1.8)	1 (0.2)	7 (1.2)
Severe	1 (0.2)	No observations	1 (0.6)

Table 2 shows that there was evidence of crude associations with probable comorbid depression/anxiety, with the following factors: currently in school, being unemployed, living in a household on two or more social protection programs and, to a lesser extent, attaining post-secondary education. These were retained for further multivariable analysis with common determinants such as age and sex, as shown in the same table. Living in a household on two or more social protection programs remained independently associated with comorbid depression/anxiety. Borderline independent associations were observed for being currently in school and being an older adolescent. These were fitted in a final parsimonious multivariable model (Table 3). Independent associations with comorbid depression/anxiety were noted for living in a household on two or more social protection programs and being currently in school. Independent associations with probable depression were also confirmed for being an older adolescent, currently in school and living in a household on two or more social protection programs. Being an older adolescent and living in a household on two or more social protection programs was an independent determinant of probable anxiety.

**Table 2.** Sample characteristics and association of sociodemographic/socioeconomic factors with risk of probable depression and anxiety among young people in three ecological zones of Ghana Table 2. long description.

Variable	Young people	Anxiety	Depression	Comorbid depression and anxiety				
	*N* (^a^%)	*n* (%)	*n* (%)	*n* (%)	Univariate RR (95% CI)	*p*-value	Adjusted RR_1_ (95% CI)	*p*-value
Overall	602 (100)	36 (5.9)	52 (8.6)	17 (2.8)				
Determinant								
**Sex**								
Male	300 (49.8)	14 (4.7)	17 (5.6)	4(1.3)	1		1	
Female	302 (50.2)	22 (7.3)	35 (11.6)	13 (4.3)	3.23 (1.06–9.79)	0.03	2.33 (0.73–7.46)	0.15
**Age (years)**								
13–16	132 (21.9)	6 (4.6)	7 (5.4)	1 (0.8)	1		1	
17–19	206 (34.2)	7 (3.4)	17 (8.3)	3 (1.5)	1.92 (0.20–18.29)	0.04	1.21(0.14–10.36)	0.98
20–24	264 (43.9)	23 (8.7)	28(10.6)	13 (4.9)	6.50 (0.86–49.16)		1.23(0.14–10.36)	
**Education**								
No education	28 (4.7)	2 (7.1)	2 (7.14)	1 (3.6)	1	0.01	1	0.35
Basic	285 (47.3)	14(4.9)	19 (6.7)	3 (1.1)	0.29 (003–2.74)		0.43 (0.04–4.49)	
Secondary	175 (29.1)	9 (5.1)	11(6.3)	4 (2.3)	0.64 (0.07–5.52)		0.70 (0.08–6.23)	
Post secondary	114 (18.9)	11 (9.7)	20 (17.5)	9 (7.9)	2.21 (0.29–16.73)		1.69(0.19–14.92)	
**Ecological belt**								
Savannah	176 (29.0)	16 (9.1)	16 (9.1)	6 (3.4)	1			
Middle	226 (38)	10 (4.4)	25 (11.1)	8 (3.5)	1.04 (0.37–2.94)	0.40		
Coastal	200 (33.0)	10 (5.0)	11 (5.5)	3 (1.5)	0.44 (0.11–1.73)			
**Schooling status**								
Not in school	198 (32.9)	27 (13.6)	33 (16.7)	13 (6.6)	1		1	
Currently in school	404 (67.1)	9 (2.2) (	19 (4.7)	4 (1.0)	6.63 (2.18–20.08)	<0.001	3.23(0.78–13.41)	0.11
**Employment**								
Unemployed	483 (80.2)	21 (4.4)	32 (6.6)	8 (1.7)	0.48 (0.14–1.65)	0.243	1.06 (0.32–3.53)	0.92
Employed	119 (91.7)	15 (12.6)	20 (16.8)	9 (7.6)	1		1	
**Social protection**								
Not enrolled	186 (30.9)	7 (3.8)	15 (8.1)	3 (1.6)	0.48 (0.14–1.65)	0.24		
Enrolled	416 (69.1)	29 (7.0)	37 (8.9)	14 (3.4.2)	1			
**Number of social protection programs**								
No program	186 (30.9)	7 (3.7)	15 (8.1)	3 (1.6)	1	0.0005	1	0.40
One program	309 (51.3)	9 (2.9)	17 (5.5)	4 (1.3)	0.80 (0.18–3.55)		1.10 (0.24–5.12)	
Two+ programs	107 (17.8)	20 (18.7)	20 (18.7)	10 (9.3)	5.79 (1.63–20.59)		3.25 (0.24–5.12)	

*Note:* RR_1_ = adjusted for each socioeconomic/demographic factor with *p* < 0.1 in univariate models.

a May not total up to 100% because of rounding.

**Table 3.** Final multivariable model of independent determinants of probable anxiety, depression and comorbid depression/anxiety Table 3. long description.

Variables	Anxiety	*p*-value	Depression	*p*-value	Comorbid depression and anxiety	*p*-value
	Adjusted RR_2_ (95% CI)		Adjusted RR_2_ (95% CI)		Adjusted RR_2_ (95% CI)	
**Sex**						
Male	1		1			
Female	1.38 (0.72–2.66)	0.33	1.76 (1.01–3.08)	0.05	2.87 (0.90–9.08)	0.07
**Age (years)**						
13–16	1	0.51	1	0.48	1	0.56
17–19	0.55 (0.21–1.47)		1.10 (0.5–2.42)		1.52 (0.16–14.20)	
20–24	0.68 (0.27–1.76)		0.76 (0.32–1.79)		2.61 (0.29–23.32)	
**Schooling status**						
Not in school	1		1		1	
Currently in school	4.76 (2.04–11.12)	<0.001	3.02 (1.61–5.69)	0.001	0.93 (0.78–10.10)	0.11
**Number of programs**						
No program	1	0.0003	1	0.02	1	0.03
One program	0.98 (0.36–2.6)		0.79 (0.41–1.54)		0.93 (0.21–4.08)	
Two+ programs	3.63 (1.57–8.4)		1.93 (1.00–3.71)		3.70 (1.00–13.66)	

*Note:* RR_2_ = adjusted for each socioeconomic/demographic factor with *p* < 0.05 in multivariable model.

## Discussion

We set out to characterize the prevalence and correlates of probable comorbid depression and anxiety among young people aged 13–24 years across high-risk populations in three ecological zones in Ghana. Our data suggest that across the three ecological zones of Ghana, 3 in 100 young people experience probable comorbid depression and anxiety, and this prevalence is similar to what is seen in Africa (Jorns-Presentati et al., 2021) and globally (Racine et al., 2021). Factors related to social and economic disadvantages, along with school attendance, are potential determinants of comorbid depression and anxiety.

The prevalence estimates of common mental health conditions among adolescents/young people vary across Africa and other low- and middle-income countries. While some studies in Asia estimate the prevalence of depression as ranging from 21% to 98% in school-based and 18% to 88% in nonschool-based settings, our prevalence estimate of 8% is similar to pooled estimates of mental distress among adolescents in Africa, and global estimates reported within the context of COVID-19. Our estimates are, however, lower than those reported in previous studies in Ghana (Amadu et al., 2024; Obeng-Okon et al., 2024). Choice of outcome measure (Fisher et al., 2012) and study setting (Mudunna et al., 2025) are likely to have a strong influence on observed prevalence.

The cross-sectional determinants of probable comorbid depression and anxiety provide an insight into the social context in which this condition is likely to be found among young people, but do not permit causal inferences. This notwithstanding, our finding suggests an independent association between possible beneficiaries of social protection programs and increased risk of comorbid depression and anxiety. This holds the same for probable depression and probable anxiety as independent conditions. The increased risk of comorbid depression and anxiety among young people in households benefiting from more than one social support program highlights an essential psychosocial gap in the design and implementation of social protection programs for poor and vulnerable populations. Social protection programs should be structured in a way that fosters resilience and provides a safety net for poor and vulnerable households. Some studies suggest that social protection schemes and youth development programs that are implemented without coordination and integration could contribute to stress or unmet expectations in low- and middle-income countries (Omilola, 2014; Niedzwiedz et al., 2016). Social protection strategies aim to reduce poverty and inequality among poor and vulnerable families in different countries (Omilola, 2014; Ghana National Social Protection Policy, 2015; Niedzwiedz et al., 2016; United Nations Development Programme, 2016). By its definition, it should alleviate individuals from poverty with resultant effects on health and well-being (Omilola, 2014; Ghana National Social Protection Policy, 2015). Our study revealed contrary but important findings that adolescents in households with more than one social protection program were at an increased risk of probable comorbid depression/anxiety. The findings support the notion that the level of baseline poverty is an important moderator of the effectiveness of social protection interventions. Being enrolled in multiple social protection programs often signals severe poverty and chronic food insecurity, both of which are well-established structural determinants of poor adolescent mental health (Lund et al., 2010; Ofori-Atta et al., 2010; Patel et al., 2018; World Bank Group, 2018). Thus, multiple social support engagements, as indicated in this study, may be insufficient in meeting the expectations of young people. While this interpretation is supported by our data, we acknowledge possible residual confounding, and the observed association should not be interpreted as evidence that social protection increases risk. Rather, it may reflect the concentration of social protection within households facing entrenched and multidimensional disadvantage that is itself strongly associated with poor youth mental health. Furthermore, even though school environments are generally expected to be protective, this study found that current school attendance was linked to a higher risk of probable depression or anxiety. These issues may be due to academic pressure, social exclusion or a lack of accessible mental health support services in schools (Melton et al., 2016; Schulte-Körne, 2016). This aligns with findings from studies in Ghana (Salk et al., 2017) and other African countries that highlight the impact of academic demands, peer-related stress, corporal punishment and insufficient psychosocial support in schools as major sources of mental distress (Salk et al., 2017; Mridha et al., 2021). Globally, research has similarly identified school-related stress as a key factor contributing to youth mental health problems (Suldo et al., 2006; Salk et al., 2017; Mridha et al., 2021).

Contrary to what is commonly reported in the extant literature, our data did not support sex, age or education as independently associated with the risk of comorbid depression and anxiety (Salk et al., 2017; Mridha et al., 2021; WHO, 2022). In contrast, our study found a higher risk of probable depression among younger adolescents aged 13–19 years. This is consistent with research findings in Uganda, which reported that early adolescence is a critical period for the onset of emotional disorders due to biological, cognitive and social transitions (Kinyanda et al., 2011; Sawyer, 2012).

### Strengths and limitations

Our study has several strengths. Our sample size is significantly larger than similar studies in LMICs. The study employed a subnational population-based sample, thus potentially representative of the epidemiology of depression and anxiety among young people in high-risk areas of Ghana. We also used an outcome measure with established construct validity among young people in Ghana, as demonstrated in prior research (Anum et al., 2019). Our study also had some limitations. The cross-sectional design, while appropriate for establishing prevalence estimates, is unable to ascertain causality. This notwithstanding, the study offers important hypothesis-generating insights into the risk factors for depression and anxiety among young people in Ghana. We also acknowledge a potential limitation in our definition of caseness arising from the cutoffs used, as these have not been specifically validated for young people in Ghana. While this is an important consideration, we note that our use of cutoffs derived from a South African study is consistent with established practice in global mental health. In contexts where locally validated thresholds are unavailable, the field supports the pragmatic use of the best available evidence, guided by considerations of geographical proximity and cultural comparability (Patel et al., 2018; WHO, 2022). We also note that the range of potential determinants examined in this study is not exhaustive. We had limited information regarding other key psychosocial and behavioral risk factors, such as the home environment (Egan et al., 2008), bullying experience (Bhuyan and Manjula, 2017), sleep quality (Henrich et al., 2023) and alcohol/substance use (Magidson et al., 2017).

## Conclusion

The findings reaffirm the social–ecological nature of mental health among adolescents, as theorized by Bronfenbrenner (Bronfenbrenner, 1979) and operationalized in global frameworks such as the Lancet Commission on Adolescent Health and Wellbeing (Patton et al., 2016). Individual factors (such as age), institutional factors (such as schooling) and service-level factors (such as program participation) all interplay to shape young people’s experiences of comorbid depression and anxiety.

The burden of comorbid depression and anxiety reported in this study suggests the need for urgent multisectoral intervention. While international efforts like the WHO’s Mental Health Gap Action Programme (mhGAP) (World Health Organization, 2020) advocate task-sharing and school facility-based delivery of mental health care in LMICs, this study emphasizes the importance of addressing social determinants such as poverty in such programs.

## Data Availability

All data for this study can be accessed upon request to the lead author at bweobong@youku.ca

## List of abbreviations

aRR: adjusted relative risk
cDA: combined depression and anxiety
CI: confidence interval
COVID: coronavirus disease
CYP: children and young people
EA: enumeration area
GAD-7: Generalized Anxiety Disorder
GSHS: Global School-based Health Survey
LMIC: low- and middle-income countries
MhGAP: Mental Health Gap Action Programme
PHQ-9: Patient Health Questionnaire
SSA: Sub-Saharan Africa
UNICEF: United Nations Children’s Fund
WHO: World Health Organization
YP: young people

## Long descriptions

### Table 1. Long description

The table is titled Severity of mental health outcomes and contains four columns. The first column lists the Severity category. The subsequent three columns provide the count n and percentage for Depression, Anxiety, and Comorbid depression-anxiety, each with a total sample size N = 602.

* None: Depression 312 (51.3 percent), Anxiety 392 (65.1 percent), Comorbid 236 (39.2 percent).

* Mild: Depression 238 (39.5 percent), Anxiety 174 (28.9 percent), Comorbid 259 (43.0 percent).

* Moderate: Depression 40 (6.6 percent), Anxiety 35 (5.8 percent), Comorbid 98 (16.3 percent).

* Moderately severe: Depression 11 (1.8 percent), Anxiety 1 (0.2 percent), Comorbid 9 (1.5 percent).

* Severe: Depression 1 (0.2 percent), Anxiety No observations, Comorbid No observations.

Navigate back to Table 1..

### Table 2. Long description

The table presents data for 602 young people. Overall, 5.9 percent have anxiety, 8.6 percent have depression, and 2.8 percent have comorbid depression and anxiety.

* Sex: Females show a higher risk for comorbid conditions (4.3 percent) compared to males (1.3 percent), with a univariate R R of 3.23.

* Age (years): The 20 to 24 age group has the highest prevalence of comorbid conditions at 4.9 percent, compared to 0.8 percent for ages 13 to 16.

* Education: Post-secondary education correlates with the highest comorbid rate at 7.9 percent, while basic education is the lowest at 1.1 percent.

* Ecological belt: The Middle belt has the highest depression rate (11.1 percent), while the Savannah belt has the highest anxiety rate (9.1 percent).

* Schooling status: Those not in school have significantly higher rates of anxiety (13.6 percent) and depression (16.7 percent) compared to those currently in school (2.2 percent and 4.7 percent respectively).

* Employment: Employed individuals show higher comorbid rates (7.6 percent) than unemployed individuals (1.7 percent).

* Social protection: Those enrolled in programs have a comorbid rate of 3.4 percent. When looking at the number of programs, those in two plus programs have a significantly higher comorbid rate of 9.3 percent compared to 1.6 percent for those in no programs, with a univariate R R of 5.79.

Navigate back to Table 2..

### Table 3. Long description

The table presents adjusted Relative Risk R R sub 2 and 95 percent Confidence Intervals C I for three conditions across four variables.

* Sex: Male is the reference group (1). For Females, the R R sub 2 is 1.38 for Anxiety (p = 0.33), 1.76 for Depression (p = 0.05), and 2.87 for Comorbid conditions (p = 0.07).

* Age in years: 13 to 16 is the reference group (1). For ages 17 to 19, R R sub 2 is 0.55 for Anxiety, 1.10 for Depression, and 1.52 for Comorbid. For ages 20 to 24, R R sub 2 is 0.68 for Anxiety, 0.76 for Depression, and 2.61 for Comorbid. Overall p-values for age are 0.51, 0.48, and 0.56 respectively.

* Schooling status: Not in school is the reference group (1). Currently in school shows a significant increase for Anxiety (R R sub 2 = 4.76, p < 0.001) and Depression (R R sub 2 = 3.02, p = 0.001), but not for Comorbid (R R sub 2 = 0.93, p = 0.11).

* Number of programs: No program is the reference group (1). One program shows R R sub 2 values below 1. Two plus programs show a significant increase for Anxiety (R R sub 2 = 3.63), Depression (R R sub 2 = 1.93), and Comorbid (R R sub 2 = 3.70), with overall p-values of 0.0003, 0.02, and 0.03 respectively.

Navigate back to Table 3..
