# Supplementary material for: Prevalence and correlates of probable anxiety and depression among young people: A subnational cross-sectional study in Ghana
Source: Glob Ment Health (Camb). 2026 Jul 7;13:e159. doi: 10.1017/gmh.2026.10270 (PMC13433327; doi:10.1017/gmh.2026.10270)
Supplement: Peer review history

# Reviewer report: Review: Prevalence and correlates of probable anxiety and depression among young people: A subnational cross-sectional study in Ghana — R0/PR2

**DOI:** 10.1017/gmh.2026.10270.pr2

**Authors:** Malay Mridha

• Introduction: Please justify the need for generating evidence of correlates of anxiety and depression.

• Methods: Clarify the reason behind the selection of a subnational sample. Please justify why 20% prevalence of depression and anxiety was assumed and 1.8 design effect was chosen. As logistic regression was uses, the association estimates should be reported as adjusted odds ratio not adjusted relative risk. Justify the p-value cut-off of <0.1 for selection of variables for the final multivariable model. Since PHD-ADS was used, please elaborate the cut-offs used for the selection of outcome variable. If >9 cut-off was used, it will combine mild-moderate-severe depression/anxiety. This approach is problematic as it is prone to the loss of statistical power, masking of non-linear relationship etc. Therefore, ordinal logistic regression or multinomial logistic regression will be better approach. Moreover, logistic regression is suitable for rare outcomes. As the prevalence of outcome variable is >10%, the authors may also report prevalence ratio instead of odds ratio. The authors also need to justify the selection of explanatory variables for the logistic regression models.

• Results: Revise the results as suggested in the method section. Report the prevalence of no/mild/moderate/severe depression/anxiety to develop more insight. The crude model can be added as a supplementary material.

• Discussion: Though the authors tried to discuss the association between access to social protection programs and depression/anxiety co-morbidity, underlying poverty may be the associated with depression-anxiety co-morbidity not the access. Please discuss this aspect too.

• Conclusion: Revise conclusion section based on the results only.

# Reviewer report: Review: Prevalence and correlates of probable anxiety and depression among young people: A subnational cross-sectional study in Ghana — R0/PR3

**DOI:** 10.1017/gmh.2026.10270.pr3

**Authors:** Anum Nisar

**Affiliations:** University of Liverpool Faculty of Health and Life Sciences, United Kingdom of Great Britain and Northern Ireland

Thank you for the opportunity to review this manuscript. This study addresses an important and under-researched topic, namely the prevalence and correlates of probable depression and anxiety among young people in Ghana. The focus on youth mental health in a low- and middle-income country context is highly relevant, and the use of established screening instruments (PHQ-9 and GAD-7), a relatively large sample size, and inclusion of multiple ecological zones are important strengths. The findings have potential relevance for informing youth mental health policies and interventions in Ghana and similar settings.

However, several methodological and interpretative issues require clarification and revision before the manuscript can be considered for publication.

Major Comments

1. Sampling strategy and representativeness

A key concern relates to the sampling approach and representativeness of the study population. The manuscript indicates that participants were selected based on belonging to marginalised or vulnerable groups (e.g., low-income populations, displaced individuals, and other disadvantaged populations). This raises concerns regarding selection bias and limits the extent to which findings can be generalised to the broader youth population in Ghana.

Despite this, the manuscript refers to the study as “population-based” and discusses implications at the national level. The authors should clarify whether the sample is representative of the general youth population or a high-risk subpopulation. The limitations related to sampling and generalisability should be explicitly acknowledged, and interpretations and conclusions should be revised accordingly.

2. Statistical analysis and reporting of relative risks

The manuscript reports adjusted relative risks (aRR) derived from logistic regression models. Logistic regression typically produces odds ratios unless alternative methods (e.g., marginal standardisation, modified Poisson regression, or log-binomial regression) are used.

The authors should provide a clearer description of the analytical approach used to derive relative risks, including whether marginal standardisation or other appropriate techniques were applied. This clarification is necessary to ensure transparency and accuracy of the reported estimates.

3. Validity of screening tool cut-offs in this population

The manuscript notes that criterion validity of PHQ-9 and GAD-7 cut-offs has not been established among adolescents in Ghana, yet applies cut-offs derived from other contexts. This may affect the accuracy of prevalence estimates.

The authors should provide a stronger justification for the selected cut-offs and discuss the potential impact of using non-validated cut-offs in this population. This limitation should be clearly acknowledged in the limitations section.

4. Interpretation of associations, particularly social protection programme participation

The finding that participation in multiple social protection programmes is associated with increased risk of depression and anxiety is important. However, given the cross-sectional design, causal interpretation is not possible. This association may reflect underlying socioeconomic disadvantage rather than an effect of programme participation itself.

The authors should avoid causal language and ensure that interpretations reflect the observational nature of the study. Alternative explanations, including confounding and reverse causation, should be discussed more explicitly.

5. Translation and administration of screening instruments

The manuscript indicates that the PHQ-9 and GAD-7 were administered in English with flexibility to translate into local languages. However, no details are provided regarding translation procedures, standardisation, or quality assurance.

The authors should clarify whether formal translation, back-translation, or standardisation procedures were used and how consistency across interviewers and languages was ensured.

Minor Comments

The discussion would benefit from more balanced interpretation of findings, particularly in relation to study limitations.

Some statements in the manuscript may overstate the generalisability of the findings and should be revised to reflect the study design and sampling limitations.

The limitations section should be expanded to more fully acknowledge methodological constraints, including measurement and sampling limitations.

Minor grammatical and typographical errors should be corrected throughout the manuscript.

# Editor report: Recommendation: Prevalence and correlates of probable anxiety and depression among young people: A subnational cross-sectional study in Ghana — R0/PR4

**DOI:** 10.1017/gmh.2026.10270.pr4

**Affiliations:** Department of Primary Care & Mental Health, University of Liverpool, UK, United Kingdom of Great Britain and Northern Ireland

Dear Authors 

We have received comments from the reviewers and in light of that we recommend major revision to your manuscript. 

Please revise your manuscript for us to consider it for publication. 

Regards

Siham

# Editor report: Decision: Prevalence and correlates of probable anxiety and depression among young people: A subnational cross-sectional study in Ghana — R0/PR5

**DOI:** 10.1017/gmh.2026.10270.pr5

**Affiliations:** London School of Hygiene & Tropical Medicine, United Kingdom of Great Britain and Northern Ireland

No accompanying comment.

# Editor report: Recommendation: Prevalence and correlates of probable anxiety and depression among young people: A subnational cross-sectional study in Ghana — R1/PR7

**DOI:** 10.1017/gmh.2026.10270.pr7

**Affiliations:** Department of Primary Care & Mental Health, University of Liverpool, UK, United Kingdom of Great Britain and Northern Ireland

Dear Authors 

We have now reviewed the major revisions you have carried out to the manuscript, in light of the comments. Your manuscript has now been accepted for publication. Congratulations. 

Our team will be inform you of the next steps. 

Regards

Siham

# Editor report: Decision: Prevalence and correlates of probable anxiety and depression among young people: A subnational cross-sectional study in Ghana — R1/PR8

**DOI:** 10.1017/gmh.2026.10270.pr8

**Affiliations:** London School of Hygiene & Tropical Medicine, United Kingdom of Great Britain and Northern Ireland

No accompanying comment.
